# In Nasal Mucosal Secretions, Distinct IFN and IgA Responses Are Found in Severe and Mild SARS-CoV-2 Infection

**DOI:** 10.3389/fimmu.2021.595343

**Published:** 2021-02-25

**Authors:** Juliana de Melo Batista dos Santos, Camila Pereira Soares, Fernanda Rodrigues Monteiro, Ralyria Mello, Jonatas Bussador do Amaral, Andressa Simões Aguiar, Mariana Pereira Soledade, Carolina Sucupira, Milena De Paulis, Juliana Bannwart Andrade, Flavia Jaqueline Almeida, Marco Aurélio Palazzi Sáfadi, Luciana Becker Mau, Jamile Menezes Brasil, Theresa Ramalho, Flávio V. Loures, Rodolfo Paula Vieira, Edison Luiz Durigon, Danielle Bruna Leal de Oliveira, André Luis Lacerda Bachi

**Affiliations:** ^1^Post-graduation Program in Sciences of Human Movement and Rehabilitation, Federal University of São Paulo, São Paulo, Brazil; ^2^Laboratory of Clinical and Molecular Virology, Department of Microbiology, Institute of Biomedical Science of University of São Paulo, São Paulo, Brazil; ^3^Ear, Nose and Throat (ENT) Lab, Department of Otorhinolaryngology, Federal University of São Paulo, São Paulo, Brazil; ^4^Method Faculty of São Paulo, São Paulo, Brazil; ^5^Infection Control Service, São Luiz Gonzaga Hospital of Santa Casa de Misericordia os São Paulo, São Paulo, Brazil; ^6^Infection Control Service and Epidemiological Hospital Nucleo, Municipal Children's Hospital Candido Fontoura, São Paulo, Brazil; ^7^Department of Pediatrics, School of Medicine, University Hospital, University of São Paulo, São Paulo, Brazil; ^8^Department of Pediatrics, Santa Casa de São Paulo School of Medical Sciences, São Paulo, Brazil; ^9^Infection Control Service and Epidemiological Hospital Nucleo, Menino Jesus Municipal Hospital, São Paulo, Brazil; ^10^Department of Immunology, Institute of Biomedical Sciences, University of São Paulo, São Paulo, Brazil; ^11^Institute of Science and Technology, Federal University of São Paulo, São Paulo, Brazil; ^12^Post-graduation Program in Bioengineering and Biomedical Engineering, Universidade Brasil, São Paulo, Brazil; ^13^Brazilian Institute of Teaching and Research in Pulmonary and Exercise Immunology (IBEPIPE), Sao Jose dos Campos, Brazil; ^14^Scientific Platform Pasteur University of São Paulo, São Paulo, Brazil; ^15^Post-graduation Program in Health Science, University of Santo Amaro, São Paulo, Brazil

**Keywords:** SARS-CoV-2, cytokine-immunological terms, respiratory, viruses, mucosal immmunity, secretory immunoglobulin A

## Abstract

Likely as in other viral respiratory diseases, SARS-CoV-2 elicit a local immune response, which includes production and releasing of both cytokines and secretory immunoglobulin (SIgA). Therefore, in this study, we investigated the levels of specific-SIgA for SARS-CoV-2 and cytokines in the airways mucosa 37 patients who were suspected of COVID-19. According to the RT-PCR results, the patients were separated into three groups: negative for COVID-19 and other viruses (NEGS, *n* = 5); negative for COVID-19 but positive for the presence of other viruses (OTHERS, *n* = 5); and the positive for COVID-19 (COVID-19, *n* = 27). Higher specific-SIgA for SARS-CoV-2, IFN-β, and IFN-γ were found in the COVID-19 group than in the other groups. Increased IL-12p70 levels were observed in OTHERS group as compared to COVID-19 group. When the COVID-19 group was sub stratified according to the illness severity, significant differences and correlations were found for the same parameters described above comparing severe COVID-19 to the mild COVID-19 group and other non-COVID-19 groups. For the first time, significant differences are shown in the airway's mucosa immune responses in different groups of patients with or without respiratory SARS-CoV-2 infection.

## Introduction

Although there are still many uncertainties about COVID-19, a widely accepted aspect of this disease concerns the fact that cells which express angiotensin-converting enzyme II (ACE2) receptors on their surface are susceptible to infection by the SARS-CoV coronavirus. The binding affinity of protein S and ACE2 is being considered one of the main determinants of the SARS-CoV replication rate and also the severity of the disease (1–3). Among several tissues that express ACE2, the nasopharyngeal mucosa can be highlighted (2, 3). In patients diagnosed with COVID-19, symptomatic and asymptomatic, nasal swabs appear to produce higher viral loads than throat swabs, strengthening the view that the nasal epithelium is the entry for infection and initial transmission (1). Assuming that SARS-CoV-2 behaves similarly to other respiratory viruses (4), its presence in the respiratory airways undoubtedly will elicit an immune response, which includes production and releasing of both cytokines and secretory immunoglobulin (SIgA) (5–7).

It is widely accepted that secretory immunoglobulin A represents the “first line of defense” against several pathogens by its capacity to directly inhibit the pathogens proliferation in the mucosa (8–10). According to the literature, reduced airways mucosal SIgA levels leads to an increased risk of developing upper respiratory tract infections (URTI) (11), mainly by respiratory virus (12). It is also of utmost highlight that decreased SIgA levels are closely related to the illness severity (13).

It should be emphasized that intranasal antigen exposure, for instance with influenza virus or respiratory syncytial virus, is able to elicit a significant IgA response in nasopharyngeal lymphoid tissue (NALT) by antigen-specific B-cell, recognized as IgA-secreting cells that were originated in the NALT (14).

Furthermore, there is a handful of evidence showing that nasal immunization also induces the cytokine expression in order to elicit protective immunity. In the same way, the infection by respiratory virus, such as coronavirus, can trigger an immune response and the cytokines release revealing a signature that can lead to a wider and unifying definition of functional biomarkers of this infection (15, 16). It has been observed that the respiratory infections can induce different immune response, such as Th1, Th2, and Th17 responses, in order to guarantee a protective immunity, including the induction of mucosal specific-SIgA against the pathogenic agent (15).

According to the literature, studies that aimed to evaluate the role of cytokines in the coronavirus infection were focused on the systemic analysis. However, its paramount to better understand how the importance of mucosal immune response are involved in this context. This accumulated knowledge about cytokines opens a favorable field and can provide relevant data to clarify the immune response capacity of the person involved in the coronavirus infection. Therefore, in this study, for the first time, results concerning the mucosal immune response are shown (both cytokines and SIgA) from COVID-19 patients.

## Materials and Methods

### Subjects of the Study

This study was carried out using nasopharyngeal and oropharyngeal swabs samples obtained from thirty-seven (17) patients who were suspected of being infected with SARS-CoV-2 (COVID-19) during SARS-CoV-2 outbreak in Brazil. The samples were collected in five hospitals in the São Paulo City: 1. University Hospital at the São Paulo University (HU-USP); 2. Santa Casa da Misericórdia Hospital (SCMH); 3. Hospital São Luiz Gonzaga (HSLG); 4. Infant Hospital Candido Fontoura (IHCF); 5. Municipal Pediatric Hospital Menino Jesus (MPHMJ). Regarding the classification of COVID-19 patients at different stages of the disease (mild, moderate and severe), it was followed the criteria presented in the file named “Guidelines for the Management of Patients with COVID-19” provided by the Brazilian Ministry of Health (18). It is noteworthy to clarify that this guideline is in agreement with the orientations provide by World Health Organization (19) for COVID-19. The nasopharyngeal and oropharyngeal swab samples were collected during the period of March to April 2020, from 1 to 5 days after the onset of the symptoms and they were used to perform the diagnosis of the occurrence of infection by several different types of virus, using respiratory virus panel (described below) through RT-PCR test. In agreement to the results obtained, we were able to stratify these patients in three different groups: the negative group for COVID-19 (NEGS, *n* = 5), the negative group for COVID-19 but positive for the presence of other virus (OTHERS, *n* = 5), and the positive group for COVID-19 (COVID-19, *n* = 27). Data about age, gender and clinical parameters, including the COVID-19 severity, are shown in the Table 1.

**Table 1:** Age (median with minimum and maximum values), number of women and men, and clinical symptoms presented by the patients who composed the negative, others, and COVID-19 groups.

**Characteristics**	**Patients (*****n*** **= 37)**			
	**Negative (*n* = 5)**	**Other viruses (*n* = 5)**	**COVID-19 (*n* = 27)**	***p value***
Age (year)	42.2 (22-73)*	12 (<1–32)	36.4 (10–73)*	<0.05
Women (*n*)	4	3	19	>0.05
Men (*n*)	1	2	8	>0.05
W/M ratio	4:1	1.5:1	2.4:1	>0.05
**Clinical symptoms (*****n*****)^#^**				
Fever	2	1	11	>0.05
Cough	2	2	11	>0.05
Coryza	0	2	10	>0.05
Sore throat	1	0	2	>0.05
Dyspnea	1	2	3	>0.05
Fatigue	1	0	1	>0.05
Body pain	1	1	5	>0.05
Myalgia	1	0	6	>0.05
Headache	0	0	8	>0.05
Anosmia	0	0	4	>0.05
Dysgeusia	1	0	1	>0.05
Chill	1	0	2	>0.05
Nasal congestion	1	0	1	>0.05
Bronchiolitis	0	1	0	>0.05
ARDS	0	3	3	>0.05

# *Number of individuals*.

Significance level of *p < 0 05.

All the subjects enrolled in this study signed the informed consent form previously approved by the Ethics Committee of the University of São Paulo and by the National Research Ethics Committee (number 36011220.0000.0081). It is noteworthy to highlight that both the study and all experiments were performed in accordance with the Declaration of Helsinki.

### RNA Extraction and Determination of Virus Infection by RT-PCR

Clinical Samples were extracted on the NUCLISENS® easyMag platform (bioMérieux, Massachusetts, USA) and real-time PCR performed on ABI 7300 machine utilizing the AgPath-ID One-Step RT-PCR master mix kit (Applied Biosystems Inc., USA) using a describe protocols for SARS-CoV-2, Respiratory Syncytial Virus type A and B (RSV-A/B), Human Metapneumovirus (HMPV), Parainfluenzavirus (PIVI-1-4), Adenovirus (AdV), Rhinovirus (RV), Influenza Virus type A and B (Flu- A/B) and sazonal Coronavirus type 1-4 (CoV 229E, CoV OC43, CoV NL63, CoV HKU1) (20–28).

### Determination of Secretory Immunoglobulin A (SIgA)

Secretory IgA immunoglobulin was detected by ELISA method. Briefly, 96-well plates (Corning, New York, USA) were coated with the nucleoprotein antigen nCoV-PS-Ag7 (Fapon Biotech Inc., Dongguan, China) (0.2 ug/mL in sodium carbonate–sodium bicarbonate buffer) and incubated at 37°C for 1 h. Unspecific binding of the antibodies was avoided by blocking with bovine serum (Advagen Biotech ltda, Itu, Brazil) at 37°C for 3 h. After washing three times with PBST, 100 μL of a mix of nasopharyngeal and oropharyngeal swabs was added and incubated for 1 h at 37°C. After washing three times with PBST, the bound antibodies were detected by using the following secondary antibody conjugated with horseradish peroxidase diluted 1:4,000 of goat anti-human IgA (Sigma-Aldrich Co., Deisenhofen, Germany). After incubation for 1 h at room temperature and three PBST washes, 100 μL of 3, 3′, 5, 5′- tetramethylbenzidine (Thermo Scientific, Massachusetts, USA) was added to each well and the mixture was incubated for 10 min at room temperature. The reaction was stopped by adding 0.2 N sulfuric acid to the mixture, and the optical density at 450 nm was measured.

### Determination of Cytokines

Cytokine concentrations were determined in the nasopharyngeal swab samples by ELISA test. Biomarkers were: interferon (IFN)-α and IFN-β (PBL Assay Science, NJ, USA), IFN-γ (Peprotech, NJ, USA), interleukin (IL)−37 and IL-17A (R&D System, Minneapolis, MN, USA), IL-6 and IL-10 (Invitrogen by Thermo Fisher Scientific, Vienna, Austria), IL-12p70 (Biolegend, San Diego, CA, USA) following the manufacturer's instructions. Concentration of cytokines was calculated using appropriate standard curves (following instructions from manufacturers). All correlation coefficients of standard curves were in the range of 0.95–0.99, whereas intra-assay coefficients of variance were 3–5%, and interassay coefficients of variance were 8–10%. Nasopharyngeal swab cytokine concentration values were normalized by the total protein concentration determined by the Bradford method (29).

### Statistical Analysis

All data obtained from the SIgA and cytokines analysis were initially compared with the Gauss curve and the normality for each determined by the Shapiro-Wilk test, followed by the homogeneity of variance analysis by the Levene test. Concentrations of these biological parameters in the patients groups were analyzed using Kruskal–Wallis with Dunn *post hoc* test and were presented as the median with the respective quartiles. In addition, it was performed a Pearson's correlation test. Significance was established with α risk at 5.0% level (*p* ≤ 0.05), and all the analysis was performed data using GraphPad Prism (version 8.1.2) software.

## Results

According to the data presented in Table 1, the mean age of the group named as Others was lower than in the negative and COVID-19 groups (*p* < 0.001 for both groups). In this respect, it is necessary to clarify that the patients who composed this group presented infection by different respiratory viruses, other than the SARS-CoV-2. By RT-PCR test, it was verified that one patient was infected by rhinovirus, one patient was infected by P3 or P4, RSV infected two patients, and also one patient showed co-infection by adenovirus, RSV, P3, or P4. It is well-known that these respiratory viruses often infect infants and children, so this fact corroborates our observation of lower mean age for this group. Even though the allocation of pediatric (*n* = 3) and adolescent/adults (*n* = 2) patients in the OTHER groups could be considered a limitation of the study leading a remarkable impact on the results, according to the data presented in the Supplementary Figure 1, both the cytokines and SIgA analysis in this group, separated into two subgroups based on the age [C = children and infant (*n* = 3, with ages 0.6, 3, 3.3 years), and A = adolescent and adult (*n* = 2, with ages 14 and 32 years)] did not show significant statistical differences. Based on these findings, all the results in the OTHER groups were presented together.

In relation to the proportion of women and men, and also to the clinical symptoms, the Chi-square test did not show significant differences between the groups. It is also important to mention that Table 1 shows a similar occurrence of ARDS in the groups of patients infected by SARS-Cov-2 or by other viruses.

**Figure 1:**
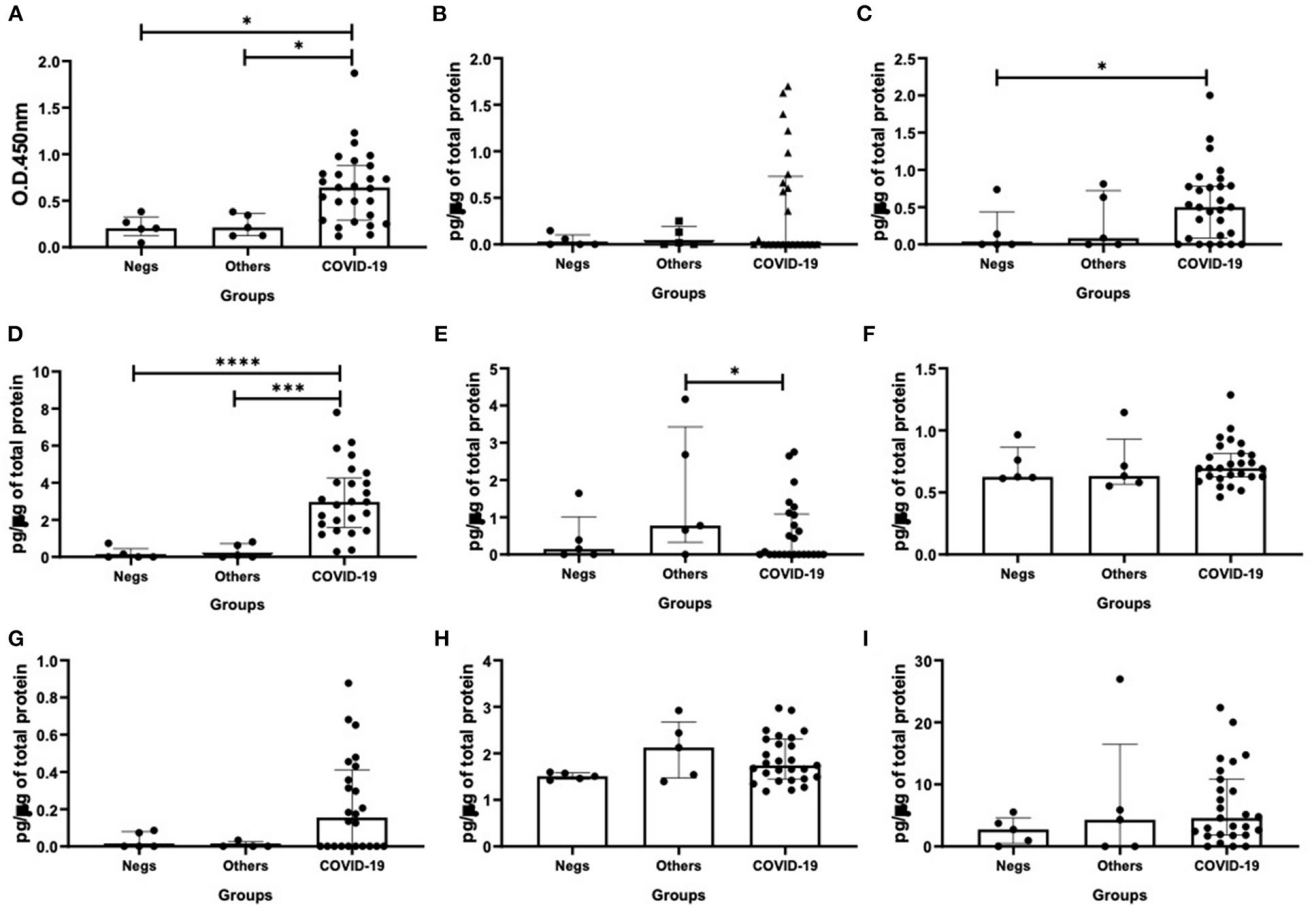
Comparison of the levels of SIgA levels **(A)**, cytokine levels of interferon (IFN)-α **(B)**, IFN-β **(C)**, IFN-γ **(D)**, interleukin (IL)-12p70 **(E)**, IL-6 **(F)**, IL-17 **(G)**, IL-10 **(H)**, and IL-37 **(I)** between negative, other viruses and COVID-19 groups. Values are presented in median and with respective quartile. Statistical analysis: Kruskal–Wallis with Dunn *post hoc* test level of significance was established at 5% (*p < 0.05; ***p < 0.001; ****p < 0.0001).

Regarding the results shown in Figure 1, as expected, the specific-SIgA levels in nasopharyngeal samples from the COVID-19 group were higher than the other patients' groups (vs. Negs - *p* = 0.02; vs. Others - *p* = 0.04, Figure 1A). In relation to the cytokine levels, higher levels of IFN-β (*p* = 0.03, Figure 1C) and IFN-γ (*p* = 0.04, Figure 1D) were observed in the COVID-19 group than the negative patients' group. Concerning the IL-12p70 levels, the group composed by patients who presented infection by other viruses showed higher levels of this cytokine than the COVID-19 group (*p* = 0.04, Figure 1E). No significant difference was found in relation to the other cytokines evaluated here.

Based on the observation that the patients composing the COVID-19 group presented differences in the illness severity, as shown in Table 1, the importance in the evaluation of biological parameters studied here in separated groups of COVID-19 patients are guarantee. Therefore, we initially distributed these patients in two subgroups: mild COVID-19 group composed by the patients who presented mild symptoms (*n* = 24), and severe COVID-19 group composed by the patients who presented ARDS (*n* = 3). In addition, the findings that some patients in the mild COVID-19 group presented levels of SIgA below the threshold, whereas others presented levels of this antibody above the threshold, allowed us to separate the mild COVID-19 group in two subgroups: mild B group (*n* = 7), which were composed by mild COVID-19 group presenting SIgA levels below the threshold, and mild A group (*n* = 17), which were composed by mild COVID-19 group presenting SIgA levels above the threshold.

**Figure 2:**
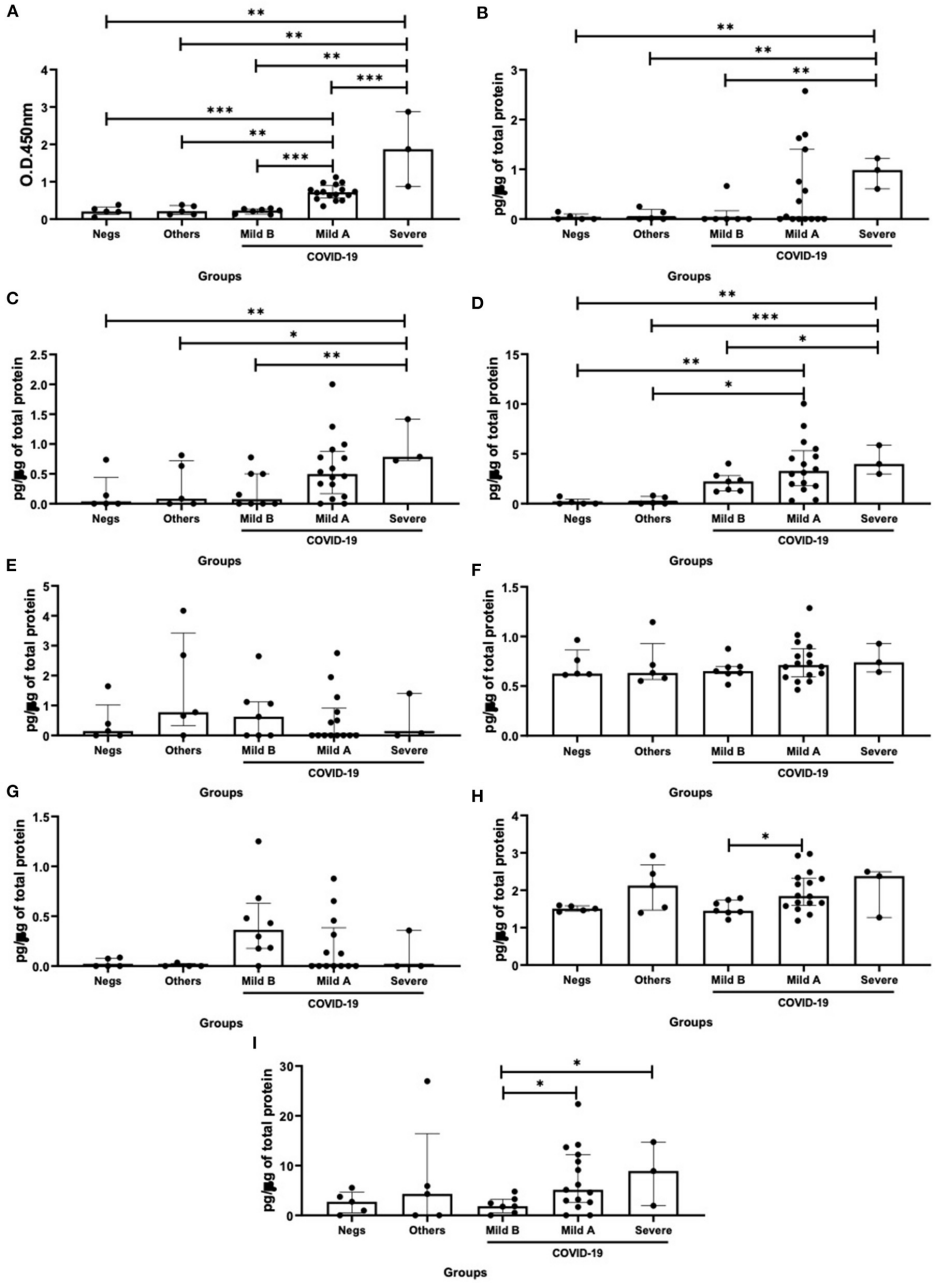
Comparison of the levels of SIgA levels **(A)**, cytokine levels of interferon (IFN)-α **(B)**, IFN-β **(C)**, IFN-γ **(D)**, interleukin (IL)-12p70 **(E)**, IL-6 **(F)**, IL-17 **(G)**, IL-10 **(H)**, and IL-37 **(I)** between negative, other viruses and mild B, mild A and severe COVID-19 groups. Values are presented in median and with respective quartile. Statistical analysis: Kruskal–Wallis with Dunn *post hoc* test level of significance was established at 5% (*p < 0.05; **p < 0.01; ***p < 0.001).

As shown in Figure 2, higher SIgA levels were found in the severe COVID-19 group than the others groups (vs. negative – *p* = 0.003; vs. others – *p* = 0.004; vs. mild B – *p* = 0.001; and vs. mild A – *p* < 0.0001). In addition, the mild A group showed increased SIgA levels as compared to the mild B (*p* = 0.0003), others (*p* = 0.002) and negative (*p* < 0.0001) groups (Figure 2A). Concerning the interferons levels, it was observed that the levels of IFN-α (Figure 2B), IFN-β (Figure 2C), and IFN-γ (Figure 2D) in the severe COVID-19 group were higher than in the mild B (*p* = 0.004, *p* = 0.009, and *p* = 0.02, respectively), others (*p* = 0.001, *p* = 0.04, and *p* = 0.0009, respectively) and negative (*p* = 0.006, *p* = 0.001, and *p* = 0.007, respectively) groups. In addition, the mild A group showed increased IFN-γ levels as compared to the others (*p* = 0.01) and negative (*p* = 0.002) groups (Figure 2D). In relation to the results about IL-10 levels, they were increased in the mild A group as compared to the mild B group (*p* = 0.04, Figure 2H). As shown in Figure 2I, higher IL-37 levels were found in the severe (*p* = 0.02) and mild A (*p* = 0.04) groups than in the mild B group.

In order to better understand how the associations between the biological parameters related to the nasopharyngeal mucosa evaluated in this study could impact in the COVID-19 disease, we carried out the correlation statistical analysis. However, it is worth clarifying that this analysis was performed only in the groups with, at least, 5 individuals. So, it was not possible to show results about the correlation in the severe COVID-19 group.

In Table 2 are shown only the results with significant correlations. Whereas, no significant correlations were found in the negative group, a positive correlation between IL-6 and IL-17 was observed in the group infected by other viruses. Concerning the correlation analysis in mild A and mild B subgroups, some similar correlations were found in both. In this respect, were observed positive correlations between IFN-α and IFN-γ, IL-6 and IL-10, as well as between IFN-γ and IL-6 and IL-10. On the other hand, different significant correlations were observed in each subgroup. In relation to the mild B subgroup, the IL-37 levels showed significant positive correlations with IFN-α, IFN-γ, IL-6, and IL-10. To the mild A subgroup, were found significant positive correlations between IFN-β and IFN-α, IFN-γ, and IL-6 as well as between IL-6 and IL-10.

**Table 2:** Significant correlations between analysis of SIgA and cytokines of mild A, mild B COVID-19 and others groups.

	**Correlation**		**Pearson *r***	***p* value**
COVID-19 Mild A (*n* = 17)	SIgA	IL-17	0.561	0.024
	IFN-α	IFN-β	0.530	0.035
	IFN-α	IFN-γ	0.689	0.003
	IFN-α	IL-6	0.829	>0.001
	IFN-α	IL-10	0.580	0.018
	IFN-β	IFN-γ	0.622	0.010
	IFN-β	IL-6	0.633	0.008
	IFN-γ	IL-6	0.771	>0.001
	IFN-γ	IL-10	0.654	0.006
	IL-6	IL-10	0.783	>0.001
COVID-19 Mild B (*n* = 7)	IFN-α	IFN-γ	0.849	0.008
	IFN-α	IL-37	0.780	0.023
	IFN-α	IL-6	0.840	0.009
	IFN-α	IL-10	0.847	0.008
	IFN-γ	IL-37	0.940	0.001
	IFN-γ	IL-6	0.965	>0.001
	IFN-γ	IL-10	0.968	>0.001
	IL-37	IL-6	0.969	>0.001
	IL-37	IL-10	0.974	>0.001
Others (*n* = 5)	IFN-α	IFN-β	0.967	0.007
	IL-6	IL-17	0.980	0.003

**Figure 3:**
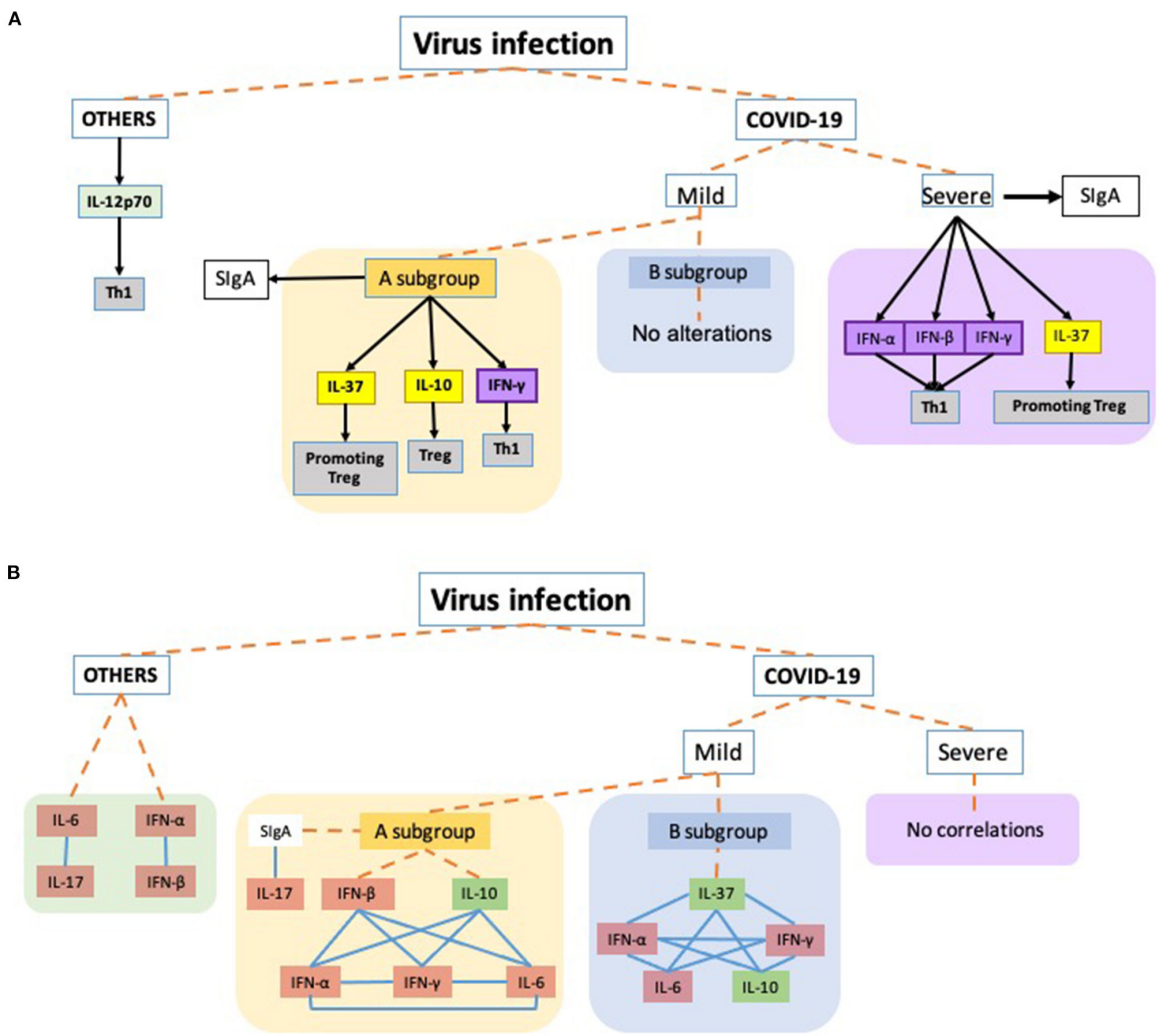
Schematic representation of the main findings of this study. Dashed orange lines virus infection to each virus groups: OTHERS (other respiratory viruses, not SARS-Cov2), COVID-19 severity (Mild: mild symptoms and Severe: acute respiratory distress syndrome) and the respective subgroups. COVID-19 Mild A subgroup was composed of patients presenting SIgA levels above the threshold and the Mild B subgroup was composed of mild COVID-19 group presenting SIgA levels below the threshold. **(A)** Black arrows toward the boxes mean that the cytokines were released by the group/subgroup. The black arrow connecting the severe subgroup to SIgA indicates a higher increase in the antibody levels than observed in the Mild A subgroup which also presented increased levels. Yellow boxes are representing anti-inflammatory cytokines involved in the T regulatory response (Treg), while the purple boxes are representing the interferons, and the gray boxes indicate the t cell profile involved in the corresponding cytokine. **(B)** Representation of correlations observed in the study. Blue lines connecting the boxes mean that the parameters presented significant positive correlations between them. Orange boxes representing the pro-inflammatory cytokines and the green boxes corresponding to anti-inflammatory cytokines.

In Figure 3, an overview of all data found in this study are shown.

## Discussion

The results of this study showed, for the first time, that COVID-19 patients presented significant differences in the mucosal immune response as compared to patients with respiratory symptoms (with or without other virus infection), and also more significant differences between cytokines and SIgA in terms of COVID-19 severity. In relation to the comparison between the groups of patients with respiratory symptoms, COVID-19 patients showed higher levels of specific-SIgA, IFN-β and IFN-γ, and lower IL-12p70 than others groups. In addition, when COVID-19 patients were analyzed based on the illness severity (mild A, mild B, and severe) it was observed that severe COVID-19 group showed increased levels of SIgA, IFN-α, IFN-β, IFN-γ, and IL-37 as compared to the others groups. Concerning the results found in the mild COVID-19 subgroups, it was found that mild A group showed higher levels of SIgA, IL-10, and IL-37 than the mild B groups, as well as increased IFN-γ as compared to the negative and others groups.

Regarding the SIgA levels, it was reported that these antibodies present in the mucosa are closely associated with the severity of viral infection and the reduction of these levels can compromise severely the local immune response (13). So, the observation that COVID-19 patients with ARDS presented higher SIgA levels can reinforce the previous literature concerning other respiratory virus. Of note, the literature demonstrate that SIgA is an important tool for early detection of infections, normally, 1 day after the infection, whereas serum IgA and IgM can be detected after 3–5 days after the infection (30). In addition, salivary IgA can putatively provide better results than serum IgA and IgM in terms of early detection for infections (30, 31). In this respect, some evidence has indicated that this type of antibody is produced mainly by IgA-secreting cells located in the nasopharyngeal lymphoid tissue, such as NALT (8, 14). Especially, the mucosal immune system present naive B and T cells and effector B and T cells (32). So, in terms of upper respiratory tract, the inhaled antigens can be recognized by the local immune system resulting in diverse immune responses, including immune tolerance (8, 12). Oropharyngeal lymphoid tissues, which include the adenoid, palatine, and lingual tonsils (Waldeyer's ring), have an essential function in airways defense (14). In order to demonstrate the importance of nasopharyngeal lymphoid tissue in humans, the classical study performed by Ogra (33) reported a significant reduction of poliovirus-specific SIgA levels in airway secretions in children submitted to tonsillectomy. It was also reported that children with tonsils presented higher antibody levels (two to five times higher) in nasopharyngeal secretions than children whose tonsils were removed (33).

Although the SIgA present a central role for the mucosal immunity, our results showing that SIgA levels were higher in ARDS group, can raise a question until now not settled: are specific SIgA to SARS-Cov-2 able to control this infection? At this point, our findings only allow us to suggest that SIgA levels may be associated with the severity of COVID-19 and not with the resolution of the infection since we were unable to assess the neutralizing capacity of this antibody, which is a limitation of this study and should be addressed in further studies. As appealing as the evaluation of SIgA could be in terms of mucosal immunity against virus infections, the analysis of cytokines in the mucosa can amplify the comprehension of the immune/inflammatory responses involved in this context. In this sense, it is noteworthy to highlight that a significant correlation was observed between SIgA levels and the cytokine IL-17 in the group of mild COVID-19.

It should be emphasized that the cytokine IL-17 is a corollary interleukin involved in the Th17 immune response. At this point, several studies have been demonstrated that Th17 presents an essential role in the mucosal immunity against, both extracellular and intracellular, pathogens (15). This immune profile can participate in the upper and lower airways mucosal immunity improving the protection together with the specific-SIgA response (15). The best proofs for this crucial role are that IL-17 neutralization impaired the mucosal protective immunity induced by nasal vaccination and that CD4+ cells secreting IL-17 were found after influenza infection (15, 34, 35). Therefore, our finding that a significant positive correlation between the levels of IL-17 and specific-SIgA for SARS-CoV-2 can reinforce the close association of these both immunological molecules in the mucosal immunity, corroborating pieces of information described above. However, concerning the betacoronavirus infection, the literature has pointed out that elevation in the IL-17 plasma levels was associated with the acute respiratory distress syndrome (ARDS) development (36). It is worthying to clarify that plasmatic IL-17, together with other pro-inflammatory cytokines, is involved in the MERS-CoV, SARS-CoV, and SARS-CoV-2 cytokine storm, a situation evidenced during the occurrence of ARDS (37, 38). These data showed a dichotomy of actions regarding the IL-17 in the COVID-19 since this cytokine has a crucial role by improving the mucosal protective immunity, but, systemically, higher levels of the same cytokine can be involved in the worst picture involved in the COVID-19. In the meantime, we observed that the IL-17 levels showed a significant correlation with IL-6 levels in the group of patients with other respiratory viruses, which was not found in the COVID-19 groups. According to the literature, the cytokine IL-6 has participation in the Th17 immune response, so this positive correlation corroborates the literature (39, 40). In a different way, the same correlation was not found in the SARS-CoV-2 infection, which can allow us to suggest that the SARS-CoV-2 infection can elicit a different way of mucosal protective immunity.

In relation to the IL-6 actions in the mucosal immune/inflammatory responses, our group has shown that the higher expression of this cytokine is a hallmark involved in the occurrence of different symptoms in the upper airways (41, 42). In addition, we also were able to demonstrate that the optimal control of IL-6 release in the mucosa by IL-10 can putatively avoid the upper the manifestation of upper airway symptoms (41, 42). Therefore, these data can corroborate our finding that the IL-6 levels were not different between all groups (negative, others, and COVID-19 groups) since the IL-10 levels obtained in this study could contribute to the mucosal inflammatory control. At this point, we would like to highlight that a significant positive correlation was verified in the mild A COVID-19 subgroup, which corroborates this effective modulation of mucosal inflammation. Take in account this idea, it would be expected that severe COVID-19 group showed imbalance between higher IL-6 levels and/or lower IL-10 levels, showing an impairment control of inflammation, which was associated with increased upper airway symptoms, as observed in our previous studies (11, 41–43). Interestingly, recent studies have demonstrated that in the cytokine storm, a situation observed in severe COVID-19, the systemic levels of IL-6 and IL-10 are increased in the same way (7, 44). However, in the literature, there is no doubt, that the levels of systemic cytokines are not reflected in the levels of mucosal cytokines, showing specific immune/inflammatory responses in these different compartments (11, 42, 45).

It is widely accepted that IL-10 is a classical anti-inflammatory cytokine (46) that acts not only modulating several inflammatory responses but also inducing CD4+ cells to acquire a specific immunological profile, such as T regulatory (Treg) (41, 42). As previously cited in the upper airway this cytokine is associated with the reduced incidence of symptoms, and also it was reported that the lack of IL-10 compromised the development of an effective immune response during influenza infection (17). Here, we found increased IL-10 levels in the mild A COVID-19 subgroup as compared to the mild B COVID-19 subgroup. This scenario can be related to the necessity to inhibit an exacerbation of inflammation in the airway's mucosa, which could lead to a dysregulated local immune response to SARS-CoV-2 infection. Corroborating this suggestion, a positive correlation was found between IL-10 and IFN-α and IFN-γ in both mild COVID-19 subgroups.

In the literature, there is a handful of data that shows the role of interferons in the coronavirus infection. The main findings evidenced that both type I interferon (IFN-α and IFN-β) and type II interferon (IFN-γ) levels raised after MERS-CoV and SARS-CoV infection, in order to induce a host defense mechanism against virus due to its strong capacity to inhibit the replication of coronaviruses (16, 47, 48). According to the review published by Jafarzadeh et al. (49), in SARS-CoV-2 infection, the IFNs response in the upper airways, in a general way, is able to drive to the viral elimination accompanied or not with mild/moderate symptoms. However, in some individuals, the SARS-CoV-2 infection can evade this antiviral IFN response, and then, spread in the body, which can elicit the exacerbated production of proinflammatory cytokines, a situation named cytokine storm. In this sense, Hadjadj et al. (50) reported that whereas in patients with severe and critical SARS-CoV-2 infection the systemic proinflammatory cytokines levels are increased, IFNs response is decreased.

It is of utmost to point out that the data presented by Hadjadj et al. (50) in which the IFNs response is significantly reduced in both severe and critical COVID-19 patients is associated with systemic evaluations and the time-point assessed was from 8 to 17 days after onset symptoms. Here, our results showing that severe COVID-19 patients presented higher levels of IFNs response in the nasal mucosa in an earlier time-point (1–5 days) can illuminate the way to improve the knowledge on the SARS-CoV-2 infection. In this sense, whereas it was documented that type I IFNs can improve not only anti-viral immunity but also evoked an inflammatory response, which is essential for respiratory mucosa protection (51, 52), it is paramount to better understand that, similarly to other pro-inflammatory cytokines, such as IL-6, higher levels of interferons in the airways mucosa becomes a trigger and sustainer of local inflammation, thus fueling a vicious-circle exacerbating mucosal inflammation (16, 53–55), which could putatively lead to a disturbs in the protective immune response in the mucosa. Particularly, in this study, higher interferon levels were observed in the severe COVID-19 group, in special, as compared to the mild B COVID-19 subgroup.

Besides the result described above, different correlations were observed between interferons and the other cytokines evaluated in the mild COVID-19 groups. In this respect, the mild A COVID-19 subgroup showed significant positive correlations between all interferons and between IL-6 and all interferons. Interestingly, IL-10 showed significant positive correlations only with IFN-α and IFN-γ, but not with IFN-β. Although these data should be analyzed with care, the lack of correlation between IL-10 and IFN-β can putatively indicate that the IFN-β release was not impacted by the IL-10 in order to guarantee an appropriate mucosal immune response against the SARS-CoV-2 infection in this group, since IFN-β, due to its stronger hydrophobicity, has a superior capacity to inhibit coronavirus replication than the other interferons (47).

Regarding the mild B COVID-19 subgroup, it was observed significant positive correlations between IFN-α and IFN-γ, and also between these interferons and IL-6 and IL-10. In addition, comparing the significant correlations observed in the mild COVID-19 groups, we highlighted above that the lack of correlations between IL-10 and IFN-β could favor the mucosal immune response in the mild A COVID-19 subgroup. However, in relation to the group mild B COVID-19 subgroup, it is possible to highlight other significant positive correlations between IL-37 and the cytokines IFN-α, IFN-γ, IL-6, and IL-10.

The cytokine IL-37 is attracting growing interest in the scientific and clinical fields due to its ability to suppress both innate and acquired immune response as well as to inhibit inflammation (53, 56, 57). Several studies have demonstrated that IL-37 can be a powerful cytokine to control the exacerbated inflammatory response in the airway's mucosa, as observed in asthma and allergic rhinitis (58–60). Indeed, the release of this cytokine in the mucosa can create a favorable environment to the maintenance of appropriated mucosal immune response. Based on these properties, we can putatively suggest that the positive correlations between IL-37 and other cytokines lead to the control of mucosal inflammatory response in the mild B COVID-19 subgroup. In the same way, the higher IL-37 levels observed in both severe and mild A COVID-19 subgroups could be involved in a mechanism of inflammatory control in the airways mucosa. The immunosuppressive signature attributed to IL-37 has been attracting attention in terms of COVID-19, since some authors have proposed that its use could minimize the inflammation associated with SARS-CoV-2 infection, including the cytokine storm (53, 57).

Beyond these very important results described above, it is important to mention that the group infected with other viruses showed higher IL-12p70 levels in comparison to the COVID-19 group. In agreement with the literature, IL-12p70 can inhibit the early virus replication, can contribute to the induction of IFN-γ, and can be involved in the activation of NK cells and T lymphocytes, especially type I T helper cells (Th1) (61, 62). Therefore, this cytokine could be involved in the activation of protective immunity in this group. Corroborating this prominent activation of a mucosal immune response, it was found a significant positive correlation between IFN-α and IFN-β, which, in accordance to described above, both of these cytokines are involved in the protection against the virus infection (16, 47, 48).

Based on these distinct patterns of cytokines released by these groups, we can suggest that there are significant differences between the immune/inflammatory response in the airway's mucosa in different groups of patients presenting or not respiratory virus infection.

In conclusion, COVID-19 infected patients display a different upper airway mucosal immune response compared with other groups of patients presenting or not respiratory virus infection, evidenced by changes in the levels of SIgA and cytokines, which present a correlation with the progression and severity of COVID-19.

## Data Availability Statement

The raw data supporting the conclusions of this article will be made available by the authors, without undue reservation.

## Ethics Statement

The studies involving human participants were reviewed and approved by Ethics Committee of the University of São Paulo and by the National Research Ethics Committee (number 36011220.0000.0081). The patients/participants provided their written informed consent to participate in this study.

## Author Contributions

JS, CSo, and AB conceived the study, analyzed the data, and together with RV, ED, and DO wrote the initial, final and revised draft of the manuscript. FM, TR, and FL participated in the planning and development of this study. RM and JoA participated in the laboratory analysis. DO, CSo, and RM: laboratory diagnosis analysis. AA, MSo, CSu, MD, JuA, FA, MSá, LM, and JB: hospital clinical evaluation, sample collection and patients clinical. ED: supervision and funding acquisition. All authors contributed to the article and approved the submitted version.

## Conflict of Interest

The authors declare that the research was conducted in the absence of any commercial or financial relationships that could be construed as a potential conflict of interest.

## Supplementary Material

Supplementary Figure 1 Comparison of the levels of SIgA **(A)** and cytokine levels **(B)** of interferon (IFN)-α, IFN-β, IFN-γ, interleukin (IL)-12p70, IL-6, IL-17, IL-10, and IL6, and IL- 37 between the volunteers of “other viruses” group separated into two subgroups according to the ages: A = adolescent and adult (*n* = 2, with ages 14 and 32 years), and C = children and infant (*n* = 3, with ages 0.6, 3, and 3.3 years). Values are presented in scatter plot graph and also with the median and interquartile range. The data were statistically evaluated by Student *t*-test with the level of significance was established at 5% (*p* < 0.05).
