# Supplementary figures and images for: In Nasal Mucosal Secretions, Distinct IFN and IgA Responses Are Found in Severe and Mild SARS-CoV-2 Infection
Source: Front Immunol. 2021 Feb 25;12:595343. doi: 10.3389/fimmu.2021.595343 (PMC7946815; doi:10.3389/fimmu.2021.595343)

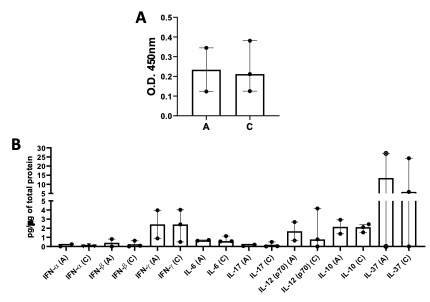

Supplement: Supplementary Figure 1 — Comparison of the levels of SIgA (A) and cytokine levels (B) of interferon (IFN)-α, IFN-β, IFN-γ, interleukin (IL)-12p70, IL-6, IL-17, IL-10, and IL6, and IL- 37 between the volunteers of “other viruses” group separated into two subgroups according to the ages: A = adolescent and adult (n = 2, with ages 14 and 32 years), and C = children and infant (n = 3, with ages 0.6, 3, and 3.3 years). Values are presented in scatter plot graph and also with the median and interquartile range. The data were statistically evaluated by Student t-test with the level of significance was established at 5% (p < 0.05). [file Image_1.JPEG]
